# Supplementary material for: PRDM9 forms a trimer by interactions within the zinc finger array
Source: Life Sci Alliance. 2019 Jul 15;2(4):e201800291. doi: 10.26508/lsa.201800291 (PMC6643046; doi:10.26508/lsa.201800291)
Supplement: Supplementary file 2 [file LSA-2018-00291_Supplemental_Data_1.docx]

# Supplementary Materials

# PRDM9 forms a trimer by interactions within the zinc finger array

Theresa Schwarz^1^, Yasmin Striedner^1^, Andreas Horner^1^, Karin Haase^1^, Jasmin Kemptner^2^, Nicole Zeppezauer^1^, Philipp Hermann^3^ and Irene Tiemann-Boege^1^

^1^Institute of Biophysics, Johannes Kepler University, Linz, Austria.

^2^Red Cross Blood Transfusion Center Upper Austria, MedCampus II, Johannes Kepler University, Linz, Austria.

^3^Institute of Applied Statistics, Johannes Kepler University, Linz, Austria.

**Table of contents**

Supplementary_Notes 3

Supplementary_Methods 17

Part 1. Preparation of biotinylated DNA 17

a. Amplification of single-Hlx1 and single-Pbx1 DNA fragments 17

b. Amplification of tandem-Hlx1-114bp DNA 17

c. Production of tandem-Hlx1-232 / 352 / 468bp 18

d. Production of tandem-Hlx1-BamHI 20

e. Amplification of unspecific DNA 20

Part 2. Cloning of murine PRDM9 constructs 20

a. Generation of eYFP-pOPIN expression vector 21

b. Generation of short PRDM9^Cst^ constructs missing certain zinc-fingers 22

c. Generation of a His-MBP-YFP-PRDM9^Dom2^-ZnF pOPIN construct 25

d. Generation of Halo-eYFP-ZnF^Cst^ 1-11 25

Part 3. Expression and semi-purification of murine PRDM9 constructs and eYFP 27

a. Bacterial expression of distinct murine PRDM9 constructs 27

b. Bacterial expression of eYFP 28

c. Preparation of murine Halo-ZnF^Cst^ 1-11 and Halo-eYFP-ZnF^Cst^ 1-11 28

d. Co-expression of eYFP-ZnF^Cst^ 2-11 and ZnF^Cst^ 2-11 29

Part 4. Electrophoretic Mobility Shift Assays 29

a. Protein Titration 29

b. Multimer Assays 30

c. Sandwich Assay 31

Supplementary_Statistical_Analysis 32

Multimer Assay I 32

Multimer Assay II 33

# Supplementary_Notes

Gel electrophoresis is a well-established method to separate molecules based on their molecular weight (MW), total charge and conformation (Orchard and May, 1993)([Orchard and May, 1993](#_ENREF_49)) by the sieving effect of the well-defined pore sizes of non-ionic agarose or polyacrylamide when applying an electrical current (Viovy, 2000).([Viovy, 2000](#_ENREF_68)) However, linear, negatively charged molecules follow another migration principle, known as the ‘reptation model’. This model proposes that the negative charge on one end is sufficient to drive the rest of the molecule and migration distance can be described as a function of the length of the linear chain. Based on this ‘reptation principle’, the MW is inversely proportional to the logarithm of the migration distance in a gel for linear, negatively charged molecules (Ferguson, 1964, Lerman and Frisch, 1982, Slater and Noolandi, 1989).([Ferguson, 1964](#_ENREF_20), [Lerman and Frisch, 1982](#_ENREF_42), [Slater and Noolandi, 1989](#_ENREF_60)) However, extremely large molecules show a migration drag known as plateau mobility and in a continuous-field electrophoresis the migration distance to MW becomes skewed (Fangman, 1978, Lerman and Frisch, 1982, McDonell et al., 1977, Viovy, 2000).([Fangman, 1978](#_ENREF_19), [Lerman and Frisch, 1982](#_ENREF_42), [McDonell et al., 1977](#_ENREF_45), [Viovy, 2000](#_ENREF_68)) Also very small fragments do not follow a size-independent mobility, known as Ogston sieving (Slater and Noolandi, 1989, Viovy, 2000).([Slater and Noolandi, 1989](#_ENREF_60), [Viovy, 2000](#_ENREF_68)) Analogous to DNA, the MW of proteins can be inferred by electrophoresis following the same reptation principle by converting proteins into negatively charged and unfolded amino acid chains with denaturing conditions and SDS (Orchard and May, 1993, Smith, 1994).([Orchard and May, 1993](#_ENREF_49), [Smith, 1994](#_ENREF_62))

At non-denaturing conditions, the mobility is also influenced by other factors other than MW, such as the charge and the conformation of the molecule (Orchard and May, 1993).([Orchard and May, 1993](#_ENREF_49)) However, the migration of truncated or extended protein variants with similar or identical charges and conformations can be compared by correlating migration distances with molecular weight (Hope and Struhl, 1987).([Hope and Struhl, 1987](#_ENREF_31)) Alternatively, the slope of the function of relative migration distances of protein standards measured in different gel concentrations can be plotted against the molecular weight of the standards (Ferguson plot) (Ferguson, 1964)([Ferguson, 1964](#_ENREF_20)) generating a curve from which the molecular weight of an unknown band can be inferred (Orchard and May, 1993).([Orchard and May, 1993](#_ENREF_49)) All these previous methods have in common that the charge and conformational components are kept constant, such that the migration distance is only a function of the molecular weight.

# Supplementary_Methods

## Part 1. Preparation of biotinylated DNA

### Amplification of single-Hlx1 and single-Pbx1 DNA fragments

**First round of PCR amplification.** In order to produce the single-Hlx1 DNA fragments for protein binding reactions, two rounds of PCR amplification were performed. In the first round of PCR 1ng/µl or 0.4ng/µl of mouse (C57BL/6 strain, *Mus musculus domesticus* origin) genomic DNA was used in combination with a primer set specific for the amplified fragment listed in Table S8 and Table S9. As polymerase either 0.015U/µl OneTaq Hot Start DNA polymerase (NEB) or 0.02U/µl Phusion U Hot Start DNA polymerase (Biozym) were used in combination with their respective buffers, 1x OneTaq Standard reaction buffer (NEB) or 1x Phusion GC buffer (Biozym), supplemented with 200µM dNTPs (Biozym) in a total of 50µl reaction. The PCR was started with an initial heating step of 94°C for 30sec, followed by 25 or 30 cycles of 94°C for 5-15sec, 61-62°C for 5-15sec, and 68°C or 72°C for 10-30sec, and a final elongation step of 5min at 68°C or 72°C (for details see Table S8). The correct size of the amplicon was validated by polyacrylamide gel electrophoresis and purified using the Wizard SV Gel and PCR Clean-Up System (Promega) according to manufacturer’s instructions.

**Second round of PCR amplification.** In the second round of PCR 10^10^ copies of the first PCR product have been used as starting material. All other reagents and conditions were kept similar to the first round of PCR. All details about PCR conditions are listed in Table S8. After checking for the correct size of the amplicon via gel electrophoresis the PCR product was treated with an Exonuclease I (NEB) digest to get rid of remaining single-stranded DNA molecules and primers as it was described in Striedner et al. (Striedner et al., 2017).([Striedner et al., 2017](#_ENREF_63)) Finally, the PCR product was purified using the Wizard SV Gel and PCR Clean-Up System (Promega) and the DNA concentration was determined using a Nanodrop 2000 instrument (Thermo Scientific) or a Denovix DS-11 FX+ Spectrophotometer (Biozym).

### Amplification of tandem-Hlx1-114bp DNA

**Hybridization of tandem-Hlx1-114bp.** In order to produce the biotinylated tandem-Hlx1-114bp fragment, complementary single-stranded oligonucleotides have been ordered as lyophilized and HPSF purified synthetic samples at the company Eurofins (sequences listed in Table S9). The oligonucleotides were dissolved in nuclease-free water (Sigma-Aldrich) to a final concentration of 150µM. Equal amounts of both strands were mixed and incubated at 98°C for 3 minutes followed by a gradual temperature decrease of 1°C/minute. To get rid of remaining single-stranded DNA fragments, an Exonuclease I (NEB) digest was performed followed by a column purification using Wizard SV Gel and PCR Clean-Up System (Promega) as described above.

**PCR amplification of Bio-tandem-Hlx1-114bp.** In a next step, the product was biotinylated by PCR amplification using 10^10^ molecules of the hybridized DNA sample and the primer set Bio-usDNA_F1 and Bio-usDNA_R1 (primer sequences are shown in Table S9). As polymerase 0.015U/µl OneTaq Hot Start DNA polymerase (NEB) was used in a 50µl reaction supplemented with 0.5µM primer, 200µM dNTPs (Biozym) and 1x OneTaq Standard reaction buffer. The initial denaturation step of 94°C for 30sec was followed by 25 cycles of 94°C 15sec, 62°C 10sec, and 68°C 10sec, and terminated by a final extension of 5min at 68°C. Finally, the amplicon was digested with Exonuclease I (NEB) and purified as described above.

### Production of tandem-Hlx1-232 / 352 / 468bp

**PCR amplification of tandem-Hlx1-232 / 352 / 468bp.** In order to produce the 232bp, 352bp and 468bp tandem-Hlx1 fragments, a series of successive PCR reactions was performed. To increase reproducibility of the final PCR, which was used to add 5’-biotin labels, the three tandem-Hlx1 fragments have been cloned into a vector so that the plasmid could be used as template.

Tandem-Hlx1-468bp. To do so, in a first step two 200 bases single-stranded ultramer fragments have been ordered at IDT (sequences listed in Table S9). The lyophilized ultramers were dissolved with nuclease-free water (Sigma-Aldrich) to a final concentration of 200µM. The ultramers contained overlapping sequences to the 5’ and 3’-end of the tandem-Hlx1-114bp DNA fragment and were fused to this sequence in an extension reaction. Therefore, 10^11^ copies of the final Bio-tandem-Hlx1-114bp PCR product (described above) were fused to 0.2µM of each of the ultramers in a 50µl reaction which was additionally supplemented by 200µM dNTPs (Biozym), 0.015U/µl OneTaq Hot Start polymerase (NEB) and 1x OneTaq Standard reaction buffer. After the initial denaturation of 94°C 30sec, only 5 cycles of 94°C 15sec, 61°C 20sec and 68°C 30sec, and a final extension of 68°C for 5min completed the protocol resulting in a 468bp long tandem-Hlx1 fragment. In a next step, this extension product was immediately used to serve as template (1µl of a 1:10^5^ dilution) for a PCR to attach biotin-tags to the 468bp long fragment. Therefore, 0.25µM of Bio-usDNA_F3 and Bio-usDNA_R3 (primer sequences listed in Table S9) were used to amplify the template using 0.01U/µl Phusion U Hot Start polymerase (Biozym) in 1x Phusion GC buffer and 200µM dNTPs (Biozym). The PCR was started with 94°C 30sec, followed by 35 cycles of 94°C 5sec, 61°C 10sec, and 72°C 8sec, and a final extension of 72°C 5min.

Tandem-Hlx1-232 and -352bp. Furthermore, 10^6^ copies of the purified amplicon Bio-tandem-Hlx1-468bp served as template to produce the tandem-Hlx1-232bp and tandem-Hlx1-352bp fragments. The primer sets 232-tandem-Hlx1_F and 232-tandem-Hlx1_R as well as 352-tandem-Hlx1_F and 352-tandem-Hlx1_R (primer sequences listed in Table S9) were used in a 50µl PCR reaction containing 0.2µM of forward and reverse primer, 200µM dNTPs (Biozym), 0.01U/µl Phusion U Hot Start polymerase (Biozym) and 1x Phusion GC buffer. Since the 5’part of the primer (lower case letters) was used to attach a biotinylated primer for the final amplification (described later), only the 3’-part of the primers could anneal to the template (upper case letters). Therefore, a two-step protocol was performed: 94°C 30sec, 10 cycles of 94°C 5s, 61°C 10sec and 72°C 4sec or 6sec (232bp or 352bp, respectively) and 25 cycles of 94°C 5sec and 72°C 14sec or 16sec (232bp or 352bp, respectively) and a final extension of 72°C 5min. The fragment sizes of all amplicons were assessed via gel electrophoresis and purified as described above.

**Prepare plasmids of** **tandem-Hlx1-232 / 352 / 468bp.** The amplicons described above were cloned in the pTZ57R/T vector via TA-cloning using the InsTAclone PCR Cloning Kit (Thermo Scientific). First, poly-A overhangs were attached to the PCR products by using 400ng of the amplicons, 200µM dNTPs (Biozym) and 0.125U/µl PeqGOLD Hot Taq DNA polymerase (Peqlab) in 1x Reaction buffer Y (Peqlab) in a total of 20µl reaction. After incubation of 10min at 68°C, the samples were purified using the Wizard SV Gel and PCR Clean-Up System (Promega). In a next step, vector and insert were ligated by adding the pure A-tailed amplicon to 50ng pTZ57R/T vector in a 3:1 molar ratio. The ligation was performed using 100U/µl T4 DNA ligase (NEB) in 1x Ligase buffer in a 20µl reaction and incubated at 16°C overnight. Then, 10µl of the cloning reaction were transformed into chemically competent *E.coli* XL1-Blue (Agilent) cells according to manufacturer’s instructions and plated on LB-agar plates (Lysogeny Broth media-based agar plates) containing 100µg/ml ampicillin and 1mg/ml X-Gal (5-Brom-4-chlor-3-indoxyl-β-D-galactopyranosid). To screen for positive clones a colony PCR was performed using 0.5µM of the primers M13_F and M13_R (primer sequences are listed in Table S9). Therefore, picked clones were used as template in a 20µl amplification reactions using 0.05U/µl OneTaq Hot Start polymerase (NEB) supplemented by 1x OneTaq Standard reaction buffer and 200µM dNTPs (Biozym). After the initial denaturation of 94°C for 5min, 30 cycles of 94°C 5sec, 61°C 10sec and 68°C 32sec followed. The reaction was completed by a final extension of 68°C for 5min. The PCR products were verified via gel electrophoresis. Selected positive clones were inoculated in 5ml LB medium containing 100µg/ml ampicillin and an overnight culture was grown allowing agitation at 37°C. 4ml of the culture were harvested by centrifugation and the plasmid was prepared using the PureYield Plasmid Miniprep System (Promega) according to manufacturer’s instructions.

**Biotinylation of tandem-Hlx1-232 / 352 / 468bp.** For a final 5’-biotinylation of the tandem-Hlx1-232bp, -352bp and -468bp fragments, PCR amplifications were performed by using 10^6^ copies of the respective plasmids in a 50µl reaction and 0.25µM of the primer combinations Bio-usDNA_F2 and Bio-usDNA_R2 (tandem-Hlx1-232bp and tandem-Hlx1-352bp) and Bio-usDNA_F3 and Bio-usDNA_R3 (tandem-Hlx1-468bp, primer sequences are listed in Table S9). As DNA polymerase 0.01U/µl Phusion U Hot Start polymerase (Biozym) in 1x Phusion GC buffer was used supplemented by 200µM dNTPs (Biozym). The PCR was started with an initial heating step of 94°C for 30sec, followed by 35 cycles of 94°C for 5sec, 61°C for 10sec, and 72°C for 6-8sec, and a final elongation step of 5min at 72°C (for details see Table S8). After checking for the correct size of the amplicons via gel electrophoresis the PCR product was treated with an Exonuclease I (NEB) digest to get rid of remaining single-stranded DNA molecules and primers, and purified using the Wizard SV Gel and PCR Clean-Up System (Promega) as described earlier.

### Production of tandem-Hlx1-BamHI

An additional tandem-Hlx1-232 fragment was produced with the tandem target sites separated by unspecific sequences also including a restriction enzyme site (Figure S2A; also see sequence in Table S9). In order to produce this fragment, a plasmid was produced with the sequence included in the Puc57-BsaI-Free vector (Biocat). Approximately 10^8^ copies of the plasmid was used as a template to amplify the tandem-Hlx1-BamHI fragment using 0.05µM of the primer pair Bio-usDNA_F2 and Bio-usDNA_R2 (primer sequences in Table S9). The reaction was supplemented with 1x Phusion HF buffer (Biozym), 200µM dNTPs (Biozym) and 0.01U/µl Phusion U Hot Start DNA polymerase (Biozym) in a total 50µl reaction volume. The PCR was started with an initial heating step of 94°C for 30sec, followed by 30 cycles of 94°C for 5sec, 61°C for 10sec, and 72°C for 5sec, and a final elongation step of 5min at 72°C (for details see Table S8). The correct size of the amplicon was validated by polyacrylamide gel electrophoresis. To remove single-stranded DNA fragments, an Exonuclease I (NEB) digest was performed followed by a column purification using Wizard SV Gel and PCR Clean-Up System (Promega) as described above.

### Amplification of unspecific DNA

In order to normalize for migration distance of unbound and bound specific DNA targets in EMSA multimer assays, unspecific DNA fragments have been included which were prepared via amplification reactions. Therefore one or two rounds of PCR amplification were performed. As template 1ng/µl or 0.4ng/µl of human (blood or saliva sample) or mouse (C57BL/6 strain) genomic DNA was used in combination with a primer set specific for the amplified fragment listed in Table S8 and Table S9. In total, four differently sized unspecific DNA molecules have been used with 75bp, 220bp, 2585bp and 4368bp. All details for PCR conditions and primer combinations can be found in Table S8. Final amplicons were assessed via gel electrophoresis, digested by Exonuclease I (NEB) and purified using the Wizard SV Gel and PCR Clean-Up System (Promega) as described earlier.

## Part 2. Cloning of murine PRDM9 constructs

For this study, different murine PRDM9 constructs have been prepared as shown in Figure 3 and Table S4. The cloning and expression of MBP-ZnF^Cst^ (used for protein titration studies, Figure 1) and MBP-YFP-ZnF^Cst^ for bacterial expression as well as for the constructs for *in vitro* expression (IVE) YFP-PRDM9^Cst^, YFP-ZnF^Cst^ and ZnF^Cst^ (used for multimer assay studies, Figure 3) is described in Striedner et al. (Striedner et al., 2017).([Striedner et al., 2017](#_ENREF_63)) For all other protein variants, the preparation is described in detail in the next sections.

### Generation of eYFP-pOPIN expression vector

**PCR amplification of eYFP-MCS.** An eYFP-containing expression vector was created based on the pOPIN-M vector from Addgene (based on an MTA with the Chancellor, Masters and Scholars of the University of Oxford, Wellington Square, Oxford, OX1 2JD, UK, Ray Owens Lab) for bacterial expression of murine PRDM9 constructs. First, eYFP was amplified flanked by a multiple cloning site (eYFP-MCS) out of 1ng peYFP-C1 vector using 5µM eYFP-NcoI-MCS_F and eYFP-MCS-HindIII_R primers (primer sequences are shown in Table S9). As polymerase 0.02 U/µl Phusion Hot Start II polymerase (Biozym) were used in a 50µl reaction in Phusion HF buffer supplemented with 0.2mM dNTPs (Biozym). The PCR cycle started with an initial heating step of 98°C for 30sec, followed by 30 cycles at 98°C for 15sec, 60°C for 15sec, and 72°C for 30sec, concluded by a final elongation step of 7min at 72°C. The correct length of the amplicon was assessed via gel electrophoresis and purified using the Wizard SV Gel and PCR Clean-Up System (Promega) according to manufacturer’s instructions. The amplicon was eluted in 30µl nuclease-free water and the DNA concentration was determined using a Nanodrop 2000 instrument (Thermo Scientific).

**Preparation of vector and insert for ligation.** Both, ~400ng of amplified eYFP-MCS and ~600ng of the pOPIN-M vector were digested using 0.5U/µl of the restriction enzymes NcoI-HF and HindIII-HF (NEB) in a 20µl reaction supplemented by 1xCutSmart buffer (NEB) for 4-5 hours at 37°C. To stop the digest the enzymes were inactivated by incubating at 80°C for 20 minutes. During the vector digest the MBP part was cut out so the remaining vector was excised from a 1% agarose gel using a clean scalpel and the linearized vector DNA as well as the digested eYFP-MCS were purified using the Wizard SV Gel and PCR Clean-Up System (Promega) according to manufacturer’s instructions. The amplicon was eluted in 30µl ddH_2_O and the DNA concentration was determined using a Nanodrop 2000 instrument (Thermo Scientific).

**Ligation of vector and insert.** 50ng of digested vector was mixed with digested insert in a molar ratio of 1:3 in a 20µl reaction containing 1x Ligase buffer and 100U/µl T4 DNA ligase (NEB) and incubated at 16°C o/n. Then 10µl of the cloning reaction was transformed into chemically competent *E.coli* XL1-Blue (Agilent) according to manufacturer’s instructions and plated on LB-agar containing 100µg/ml ampicillin. Screening for positive clones was performed by colony PCR and control restriction digests. A single colony of the positive clone was inoculated in 3ml LB medium containing 100µg/ml ampicillin and an overnight culture was grown, shaking at 37°C. 2ml of the culture was harvested by centrifugation and the plasmid was prepared using the PureYield Plasmid Miniprep System (Promega) according to manufacturer’s instructions. The integrity of the plasmid sequence was assessed by Sanger Sequencing (LGC Genomics).

### Generation of short PRDM9^Cst^ constructs missing certain zinc-fingers

In order to generate short PRDM9^Cst^ mutants, the respective parts of the murine *Prdm9* gene were cloned into the eYFP-pOPIN vector via restriction enzyme based cloning using the enzymes KpnI-HF and HindIII-HF. As it was very challenging to produce the short constructs, a lot of different steps were necessary. First, shorter parts of the repetitive ZnF array were amplified using specific primers to then clone the amplicon into a vector via TA-cloning to get a pure plasmid. With a pure plasmid as template it was possible to amplify specific regions of the ZnF array to add the proper restriction enzyme sites needed for inserting selected ZnF regions into an expression vector.

**Preparation of the eYFP-pOPIN vector for cloning.** In a 20µl reaction 500ng of eYFP-pOPIN vector were digested using 0.5U/µl of both enzymes KpnI-HF and HindIII-HF (NEB) supplemented by 1xCutSmart buffer. The digest was incubated at 37°C for 90min. To avoid vector re-circularization during ligation, the digested vector was additionally dephosphorylated using 0.2U/µl Antarctic Phosphatase (NEB) in 1xAntarctic Phosphatase buffer with an incubation at 37°C for 15min followed by an inactivation at 80°C for 20min. The linearized vector was cut out from a 0.8% agarose gel and purified using the Wizard SV Gel and PCR Clean-Up System (Promega) according to manufacturer’s instructions.

**Amplification of ZnF^Cst^ 1-11.** In a first step, the insert was amplified using 10^8^ copies of a plasmid containing the full coding sequence of murine *Prdm9^Cst^* as template and 0.25µM of each of the primers ZnFcst-1-KpnI_F and ZnFcst-11-HindIII_R (primer sequences shown in Table S9). The 50µl reaction was supplemented with Phusion GC buffer, 200µM dNTPs (Biozym) and 0.02U/µl Phusion U Hot Start polymerase (Biozym). The PCR cycle started with an initial heating step of 94°C for 30sec, followed by 10 cycles at 94°C for 5sec, 60°C for 5sec, 72°C for 28sec, and 20 cycles at 94°C for 5sec, and 72°C for 33s, concluded by a final elongation step of 5min at 72°C. The correct length of the amplicon was assessed via gel electrophoresis and cut out of the 1% agarose gel using a clean scalpel, which was then purified using the Wizard SV Gel and PCR Clean-Up System (Promega) according to manufacturer’s instructions. The amplicon was eluted in 30µl nuclease-free water and the DNA concentration was determined using a Nanodrop 2000 instrument (Thermo Scientific).

**Amplification of ZnF^Cst^ 2-11, ZnF^Cst^ 2-6 for TA-cloning.** In order to prepare the insert containing ZnF2-11 and ZnF2-6 of the murine PRDM9^Cst^ protein, the respective ZnF regions have been amplified using 10^5^ copies of a plasmid containing the full coding sequence of murine *Prdm9^Cst^* as template and 0.25µM of the primers ZnFcst-2_F and ZnFcst-11_R or 0.5µM of ZnFcst-2_F and ZnFcst-6_R, respectively (primer sequences shown in Table S9). The 50µl reaction was supplemented with Phusion GC buffer, 200µM dNTPs (Biozym) and 0.01U/µl Phusion U Hot Start polymerase (Biozym). To amplify ZnF^Cst^ 2-11, the PCR cycle started with an initial heating step of 94°C for 30sec, followed by 35 cycles at 94°C for 5sec, 64°C for 10sec, and 72°C for 15sec, concluded by a final elongation step of 10min at 72°C. To amplify ZnF^Cst^ 2-6, the PCR cycle started with an initial heating step of 94°C for 120sec, followed by 35 cycles at 94°C for 15sec, 65°C for 15sec, and 72°C for 10sec, concluded by a final elongation step of 7min at 72°C. The correct lengths of the amplicons were assessed via gel electrophoresis. The amplicon of ZnF^Cst^ 2-11 was then purified using the Wizard SV Gel and PCR Clean-Up System (Promega) according to manufacturer’s instructions.

**Purification of ZnF^Cst^ 2-6 using bead size selection.** As the ZnF^Cst^ 2-6 amplicon required a more accurate purification, a size selection using magnetic Agencourt AMPure XP beads (Beckmann Coulter) was performed. By choosing the amount of beads and therefore the amount of present polyethylenglycol, DNA of certain size will bind to the beads and is separated from others. To specifically select for the ZnF^Cst^ 2-6 amplicon, first, the PCR product was mixed with 0.6 volumes of beads and incubated at RT for 15min. The sample tube was put to a magnet for 5min to separate the beads from solution. Then, the supernatant was transferred to a fresh tube and mixed with the same volume of fresh beads. After 5min of incubation, the sample was put to the magnet for another 5min. The supernatant was discarded and the beads were washed with 200µl of 80% ethanol for two times. Finally, the selected DNA was eluted from the beads using 26µl nuclease-free water. DNA concentration was measured using the DeNovix DS-11 spectrophotometer (Biozym).

**TA-cloning of ZnF^Cst^ 2-11 and ZnF^Cst^ 2-6.** In order to get the insert into a vector, the InsTAclone PCR Cloning Kit (Thermo Scientific) was used for TA-cloning. First, A-tails were added to the inserts in a reaction of 10min at 68°C. Therefore, 400ng of amplicon was mixed with 500µM dNTPs (Biozym), 1x Reaction buffer Y and 0.125U/µl PeqGOLD Hot Taq DNA polymerase (Peqlab) in a 20µl reaction. Afterwards, the sample was purified using the Wizard SV Gel and PCR Clean-Up System (Promega) according to manufacturer’s instructions. For the ligation, 50ng of pTZ5R/T vector were mixed with a three times higher molarity of A-tailed insert. The 20µl reaction was supplemented with 1x Ligase buffer and 100U/µl T4 DNA ligase (NEB) and was incubated at 16°C o/n. The cloning procedure was continued as described above.

**Amplification of ZnF^Cst^ 2-11, ZnF^Cst^ 2-8, and ZnF^Cst^ 2-6 for RE-based cloning into the expression vector eYFP-pOPIN.** The three different ZnF constructs ZnF^Cst^ 2-11, ZnF^Cst^ 2-8, and ZnF^Cst^ 2-6 have been cloned into the eYFP-pOPIN expression vector via restriction enzyme based cloning using the enzymes KpnI-HF and HindIII-HF for further expression. Therefore, in a first step, the inserts were amplified to add the RE-recognition sites. For ZnF^Cst^ 2-11 and ZnF^Cst^ 2-8, 10^4^ copies of the plasmid ZnF^Cst^ 2-11_pTZ5R/T were used as template with 0.25µM of the primers ZnFcst-2-KpnI_F and ZnFcst-11-HindIII_R or ZnFcst-8-HindIII_R, respectively. The reaction was supplemented by 200µM dNTPs (Biozym), 1xPhusion GC buffer and 0.01U/µl Phusion U Hot Start polymerase (Biozym) in a total volume of 50µl. The PCR cycle started with an initial heating step at 94°C for 30sec, followed by 10 cycles at 94°C for 5sec, 66°C for 10sec, and 72°C for 14sec or 10sec, respectively, and 25 cycles at 94°C for 5sec and 72°C for 24sec or 20sec, respectively, concluded by a final elongation step of 5min at 72°C. ZnF^Cst^ 2-6 was amplified using 10^4^ copies of the ZnF^Cst^ 2-6_pTZ5R/T and 0.25µM of the primers ZnFcst-2-KpnI_F and ZnFcst-6-HindIII_R (all primer sequences are listed in Table S9). The 50µl reaction was supplemented by 200µM dNTPs (Biozym), 1xPhusion GC buffer and 0.01U/µl Phusion U Hot Start polymerase (Biozym). The PCR cycle started with an initial heating step at 94°C for 30sec, followed by 10 cycles at 94°C for 5sec, 64°C for 10sec, and 72°C for 10sec, and 25 cycles at 94°C for 5sec and 72°C for 20sec, concluded by a final elongation step of 5min at 72°C. The correct lengths of the amplicons were assessed via gel electrophoresis and purified using the Wizard SV Gel and PCR Clean-Up System (Promega) according to manufacturer’s instructions.

**Ligation of inserts to the eYFP-pOPIN expression vector.** First, the different inserts, ZnF^Cst^ 1-11, ZnF^Cst^ 2-11, ZnF^Cst^ 2-8, and ZnF^Cst^ 2-6, were digested in a 20µl reaction using ~600ng amplicon and 0.5U/µl of both enzymes KpnI-HF and HindIII-HF (NEB) supplemented by 1xCutSmart buffer. After incubation at 37°C for 90min, the samples were purified using the Wizard SV Gel and PCR Clean-Up System (Promega) according to manufacturer’s instructions. Afterwards, vector and insert were ligated in a 1:3 molar ratio and cloning was continued as described above. The integrity of the final plasmids were assessed with Sanger Sequencing (LGC Genomics).

**Cut out the eYFP tag.** In order to produce constructs without an eYFP tag, this part was cut out of the construct via a restriction enzyme digest. For this purpose, 500ng plasmid were digested using 0.5U/µl of the enzyme XhoI (NEB) in 1xCutSmart buffer in a total volume of 20µl. The digest was incubated at 37°C for 90min, and then loaded on a 1% agarose gel to separate the eYFP-tag from the remaining vector. The vector band was cut out of the gel and purified using the Wizard SV Gel and PCR Clean-Up System (Promega) according to manufacturer’s instructions. Finally, the linearized vector without eYFP was re-ligated by using 50ng of vector and 100U/µl T4 DNA ligase (NEB) in 1x Ligase buffer with an incubation temperature of 16°C o/n. The cloning procedure was continued as described before and the integrity of the final plasmid was assessed with Sanger Sequencing (LGC Genomics).

**Plasmid constructs.** In the following, the plasmid constructs produced as described above are shown. The self-made pOPIN vector included a His6-tag, a cMyc-tag, several restriction enzyme sites like BamHI, EcoRI, XhoI, KpnI and HindIII, an eYFP-tag (yellow) if desired and the respective insert (blue).

pOPIN_His6-cMyc-BamHI-EcoRI-XhoI-eYFP-XhoI-KpnI-*Prdm9^Cst^* ZnF1-11-HindIII_pOPIN

pOPIN_His6-cMyc-BamHI-EcoRI-XhoI-KpnI-*Prdm9^Cst^* ZnF1-11-HindIII_pOPIN

pOPIN_His6-cMyc-BamHI-EcoRI-XhoI-eYFP-XhoI-KpnI-*Prdm9^Cst^* ZnF2-11-HindIII_pOPIN

pOPIN_His6-cMyc-BamHI-EcoRI-XhoI-KpnI-*Prdm9^Cst^* ZnF2-11-HindIII_pOPIN (only used for co-expression, shown below)

pOPIN_His6-cMyc-BamHI-EcoRI-XhoI-KpnI-*Prdm9^Cst^* ZnF2-8-HindIII_pOPIN

pOPIN_His6-cMyc-BamHI-EcoRI-XhoI-KpnI-*Prdm9^Cst^* ZnF2-6-HindIII_pOPIN

### Generation of a His-MBP-YFP-PRDM9^Dom2^-ZnF pOPIN construct

**Amplification of the murine *Prdm9^Dom2^* zinc-finger region and preparation of the insert for ligation.** In order to prepare a His-MBP-YFP-PRDM9^Dom2^-ZnF protein, the insert was cloned in an eYFP-pOPIN-M vector via restriction enzyme-based cloning using NotI. In a first step, the *Prdm9^Dom2^* zinc-finger region (Exon 10) was amplified out of the plasmid *Prdm9^Dom2^*_pBAD, kindly provided by Petko M. Petkov, using 10^8^ copies of the starting template as well as 0.5µM of the primers ZnFdom2-0-NotI_F and ZnFdom2-12-NotI-HindIII_R (primer sequences are listed in Table S9). The 50µl reaction was supplemented by 200µM dNTPs (Biozym), 1xPhusion HF buffer, 0.02U/µl Phusion Hot Start II polymerase (Biozym) and 1% DMSO. The PCR cycle was started by an initial heating step of 98°C for 30sec followed by 15 cycles at 98°C for 10sec, 60°C for 15sec and 72°C for 60sec, and another 10 cycles at 98°C for 10sec and 72°C for 75sec, concluded by a final elongation step of 7min at 72°C. The integrity of the amplicon was assessed by gel electrophoresis; the band was cut out from a 1% agarose gel and purified using the Wizard SV Gel and PCR Clean-Up System (Promega) according to manufacturer’s instructions. The purified insert was digested in a 20µl reaction using 0.5U/µl of the restriction enzyme NotI-HF (NEB) in 1xCutSmart buffer for 90min at 37°C. The digestion was inactivated by incubating at 65°C for 20min and purified using the Wizard SV Gel and PCR Clean-Up System (Promega) according to manufacturer’s instructions.

**Ligation of insert and vector.** An eYFP-containing pOPIN-M vector-plasmid (preparation described in Striedner et al. (Striedner et al., 2017)) was prepared by NotI digestion and de-phosphorylation as previously described.([Striedner et al., 2017](#_ENREF_63)) 50ng of the prepared vector were mixed with a three times higher molarity of prepared *Prdm9^Dom2^*-*ZnF* insert and ligated using 100u/µl T4 DNA ligase (NEB) in 1x Ligation buffer by incubation at 16°C o/n. The cloning procedure was continued as described above.

**Plasmid construct.** In the following, the plasmid construct produced as described above is shown. It includes a His6-tag, a maltose binding protein tag (MBP), a PreScission cleavage site as well as a TEV cleavage site, a cMyc-tag, several restriction enzyme sites like KpnI, XhoI, NotI and HindIII, an eYFP-tag (yellow) and the respective insert (blue).

pOPIN_His6-MBP-KpnI-PreScission-XhoI-eYFP-XhoI-NotI-TEV-cMyc-*Prdm9^Dom2^* ZnF-NotI-HindIII_pOPIN

### Generation of Halo-eYFP-ZnF^Cst^ 1-11

In order to fluorescently tag the Halo-ZnF^Cst^ 1-11 protein construct, an eYFP tag was inserted to this plasmid by restriction enzyme based cloning with the enzymes XhoI and PvuI, producing the construct Halo-eYFP-ZnF^Cst^ 1-11. The plasmid Halo-ZnF^Cst^ 1-11 is based on the pH6HTN-His6-HaloTag-T7 vector backbone and was prepared as described in Walker et al. (Walker et al., 2015) and kindly provided by Petko M.([Walker et al., 2015](#_ENREF_69)) Petkov.

**Amplification of eYFP as insert for ligation.** The eYFP insert was produced in two rounds of PCR reactions. In the first round, eYFP was amplified using 10^8^ copies of a plasmid harbouring the eYFP gene and 0.05µM of each primer eYFP_F and eYFP_R (primer sequences listed in Table S9). The reaction was supplemented with 1x OneTaq Standard reaction buffer (NEB) 200µM dNTPs (Biozym) in a total of 50µl reaction. As polymerase 0.015U/µl OneTaq Hot Start DNA polymerase (NEB) was used. The PCR was started with an initial heating step of 94°C for 30sec, followed by 30 cycles of 94°C for 15sec, 67°C for 15sec, and 68°C for 45sec, and a final elongation step of 5min at 68°C. The correct size of the amplicon was validated by polyacrylamide gel electrophoresis and purified using the Wizard SV Gel and PCR Clean-Up System (Promega) according to manufacturer’s instructions. In the second round, 10^9^ copies of the first round pure PCR product was used and amplified using 0.05µM of each primer XhoI-eYFP_F and PvuI-eYFP_R (primer sequences listed in Table S9). The reaction was supplemented with 1x OneTaq Standard reaction buffer (NEB) 200µM dNTPs (Biozym) in a total of 50µl reaction. As polymerase 0.015U/µl OneTaq Hot Start DNA polymerase (NEB) was used. The PCR was started with an initial heating step of 94°C for 30sec, followed by 25 cycles of 94°C for 15sec, 67°C for 15sec, and 68°C for 50sec, and a final elongation step of 5min at 68°C. The correct size of the amplicon was validated by polyacrylamide gel electrophoresis and purified using the Wizard SV Gel and PCR Clean-Up System (Promega) according to manufacturer’s instructions by cutting out the correct band from a 1% agarose gel.

**Restriction enzyme digest of vector and insert.** Both, 450ng of amplified eYFP insert and 1µg of Halo-ZnF^Cst^ 1-11_pH6HTN vector plasmid, were digested using 0.4U/µl of the restriction enzymes XhoI and PvuI-HF (NEB) in a 50µl reaction supplemented by 1xCutSmart Buffer for 1 hour at 37°C. A fraction of the vector digest was purified from a 1% agarose gel and the linearized vector DNA as well as the digested eYFP were purified using the Wizard SV Gel and PCR Clean-Up System (Promega) according to manufacturer’s instructions.

**Ligation of vector and insert.** 50ng of digested vector was mixed with digested insert in a molar ratio of 1:3 in a 20µl reaction containing 1x Ligase buffer and 20U/µl T4 DNA ligase (NEB) and incubated at 16°C o/n. Then 10µl of the cloning reaction was transformed into chemically competent *E.coli* XL1-Blue (Agilent) according to manufacturer’s instructions and plated on LB-agar containing 100µg/ml ampicillin. Screening for positive clones was performed by colony PCR. A single colony of the positive clone was inoculated in 5ml LB medium containing 100µg/ml ampicillin and an overnight culture was grown, shaking at 37°C. A 4ml of the culture was harvested by centrifugation and the plasmid was prepared using the PureYield Plasmid Miniprep System (Promega) according to manufacturer’s instructions. We verified the correct sequence of the plasmid by Sanger Sequencing (LGC Genomics).

## Part 3. Expression and semi-purification of murine PRDM9 constructs and eYFP

### Bacterial expression of distinct murine PRDM9 constructs

**Expression of truncated murine PRDM9^Cst^ constructs.** In order to recombinantly express the truncated constructs of PRDM9^Cst^-ZnF, 25ng plasmid have been transformed into chemically competent Rosetta^TM^2(DE3)pLacI *E.coli* cells (Novagen, Merck) according to manufacturer’s instructions. The cells were plated onto LB-agar containing 25µg/ml chloramphenicol and 100µg/ml ampicillin and grown overnight at 37°C. The next day, s single colony was inoculated in 100ml LB (Lysogeny broth after Lennox: 5g/l yeast extract, 5g/l NaCl, 10g/l tryptone) containing 25µg/ml chloramphenicol and 100µg/ml ampicillin to grow at 37°C overnight, allowing agitation. The cells were harvested by centrifugation at 5000rpm for 5min. The supernatant growth media was discarded and replaced by fresh LB-medium containing 1mM IPTG (Isopropyl-beta-D-thiogalactopyran) and 50µM ZnCl_2_ to induce the expression. The recombinant protein was overexpressed for 7hrs at room temperature allowing agitation at 160rpm. Finally, the cells were harvested by centrifugation at 5000rpm for 10min at 4°C and the pellets were frozen at -80°C at least overnight.

**Expression of murine PRDM9-ZnF^Dom2^.** To express the MBP-YFP-ZnF^Dom2^ construct, 25ng plasmid have been transformed into chemically competent BL21-AI *E.coli* cells (Invitrogen) according to manufacturer’s instructions. The cells were plated onto LB-agar containing 25µg/ml chloramphenicol and 100µg/ml ampicillin and grown overnight at 37°C. The next day, a single colony was inoculated in 100ml LB (Lysogeny broth after Lennox: 5g/l yeast extract, 5g/l NaCl, 10g/l tryptone) containing 25µg/ml chloramphenicol and 100µg/ml ampicillin to grow at 37°C for 8hrs, allowing agitation. The cells were harvested by centrifugation at 5000rpm for 5min. The supernatant growth media was discarded and replaced by fresh LB-medium containing 1mM IPTG (Isopropyl-beta-D-thiogalactopyran), 0.2% arabinose and 50µM ZnCl_2_ to induce the expression. The recombinant protein was overexpressed overnight at room temperature allowing agitation at 160rpm. Finally, the cells were harvested by centrifugation at 5000rpm for 10min at 4°C and the pellets were frozen at -80°C at least overnight.

**Lysate preparation of bacterially expression PRDM9 constructs.** Frozen 50ml pellets were thawed and dissolved in 2500µl 1xTKZN (10mM Tris, 50mM KCl, 50µM ZnCl_2_, 0.05% NP-40, pH7.5) per 0.1g cells for washing. After centrifugation at 5000rpm at 4°C for 10min, the supernatant was discarded including all soluble proteins. The pellet was dissolved in 236µl 1xTKZN + 0.3% Sarcosyl (N-Lauroylsarcosine sodium salt) per 0.1g cells to solubilize PRDM9 and centrifuged for another 10min at 5000rpm and the supernatant was collected (SN). Then, the remaining cell pellet was dissolved in 4000µl 1xTKZN + 0.3% Sarcosyl per 0.1g cells to use it as whole-cell fraction (WC).

**Protein verification on SDS-PAGE.** All lysed protein samples were verified after recombinant expression via SDS-PAGE (data not shown). Therefore, 10-20µl of the lysate sample was mixed with 1x Laemmli buffer (10% glycerol, 2% SDS, 80mM Tris-HCl pH 6.8, 5.3% beta-mercaptoethanol, 0.06% bromophenol blue) and incubated at 95°C for 5min. To keep the fluorescent activity of eYFP-labelled proteins, those samples were incubated for 5min at room temperature. Then, the samples were loaded onto an 8% or 12% SDS-polyacrylamide gel and run at 180V until the blue dye front has run out. As protein ladder, the AllBlue Precision Plus Protein Standard (Bio-Rad) was used. In order to detect eYFP fluorescence, the gel was imaged at 510nm using the ChemiDoc™ MP imager (Bio-Rad). In addition, the protein gels were stained with Coomassie-G250 afterwards.

### Bacterial expression of eYFP

In order to express the eYFP protein as a control for FCS measurements, 50ng of the empty eYFP-pOPIN vector were transformed into chemically competent Rosetta^TM^2(DE3)pLacI *E.coli* cells (Novagen, Merck) according to manufacturer’s instructions. The cells were plated onto LB-agar containing 25µg/ml chloramphenicol and 100µg/ml ampicillin and grown overnight at 37°C. The next day, s single colony was inoculated in 100ml LB containing 25µg/ml chloramphenicol and 100µg/ml ampicillin to grow at 37°C until reaching an OD600 of 1 allowing agitation. The rest of the expression and lysis preparation was done as described above.

### Preparation of murine Halo-ZnF^Cst^ 1-11 and Halo-eYFP-ZnF^Cst^ 1-11

**Expression and lysate preparation of Halo-ZnF^Cst^ 1-11** and Halo-eYFP-ZnF^Cst^ 1-11**.** In order to produce a semi-pure protein lysate of PRDM9, ~70ng of pH6HTN-His6-HaloTag-T7 vector containing murine PRDM9^Cst^ ZnF1-11 (Walker et al., 2015), which was kindly provided by Petko M.([Walker et al., 2015](#_ENREF_69)) Petkov, or eYFP-labeled PRDM9^Cst^ ZnF1-11, were transformed into chemically competent Rosetta^TM^2(DE3)pLacI *E.coli* cells (Novagen, Merck) according to manufacturer’s instructions. The cells were plated onto LB-agar containing 100µg/ml ampicillin and grown overnight at 37°C. The next day, one single colony was inoculated in 100ml LB (Lysogeny broth after Lennox: 5g/l yeast extract, 5g/l NaCl, 10g/l tryptone) containing 100µg/ml ampicillin to grow at 37°C until OD600 = 1 by allowing agitation. The cells were harvested by centrifugation at 5000rpm for 10min at 4°C and the pellets were frozen at -80°C at least overnight. A 50ml cell pellet was thawed and dissolved in 20ml 1x cell breakage buffer (50mM Tris pH7.5, 97mM Sucrose, 100mM KCl, 0.1% NP-40, 0.1% 2-mercaptoethanol, EDTA-free protease inhibitor cocktail). After six rounds of sonication for 30 seconds with a resting on ice in between, the lysate was centrifuged for 10min at 12.000rpm and the supernatant was taken.

**Ion exchange chromatography.** To additionally semi-purify the Halo-ZnF^Cst^ 1-11 or Halo-eYFP-ZnF^Cst^ 1-11 protein lysate, 20ml soluble protein were equilibrated with 1ml SP Sepharose High Performance (GE Healthcare) for 90min at 4°C allowing agitation for proper mixing. Then, the solution was poured to a 5ml polypropylene column (Qiagen) and an elution buffer (20mM KH_2_PO_4_, 0.01% glycerol, 0.1% NP-40, 0.1% 2-mercaptoethanol, EDTA-free protease inhibitor cocktail) including a KCl gradient from 100-1000mM was used to elute the protein of interest. The majority of Halo-ZnF^Cst^ 1-11 and Halo-eYFP-ZnF^Cst^ 1-11 was eluted in the 500mM KCl fraction. To verify the samples, an SDS-PAGE followed by Coomassie staining was performed as described above (see Figure S11).

### Co-expression of eYFP-ZnF^Cst^ 2-11 and ZnF^Cst^ 2-11

In order to co-express the two constructs eYFP-ZnF^Cst^ 2-11 and ZnF^Cst^ 2-11, 50ng of each independent plasmid were transformed into Rosetta^TM^2(DE3)pLacI *E.coli* cells (Novagen, Merck) and expressed simultaneously as described above. The lysate preparation was done in the same way than for all other crude lysates described before. The concentration of the expressed proteins was verified by Western blot (Figure S7A). For this purpose, 10µl of the protein lysate was loaded on a 12% SDS-PAGE and run as described above. Afterwards, the proteins were electrophoretically transferred to a PVDF membrane (Bio-Rad) in 1x blotting buffer (25mM Tris, 192mM glycine, 0.02% SDS, 10% isopropanol, pH8.2) for 80min at 100V. After blotting, the membrane was blocked at RT for 1hr with 1x blocking solution (5% BSA in 1xTBS-T = 25mM Tris, 137mM NaCl, 2.7mM KCl, 0.05% Tween-20) followed by an incubation with anti-His-HRP antibody (Milteny Biotech) in a 1:10,000 dilution in 1xTBS-T for 1hr at RT. Finally, the membrane was washed two times with 1xTBS-T before applying the chemiluminescent reaction using the WesternBright Quantum Kit from Advansta following the manufacturer’s instructions.

## Part 4. Electrophoretic Mobility Shift Assays

The EMSA reaction mixtures, incubation and running times varied depending on the experiment but followed the general protocol outlined in Striedner et al. (Striedner et al., 2017).([Striedner et al., 2017](#_ENREF_63))

### Protein Titration

EMSA protein titration experiments have been performed by using 5nM of either biotinylated single-Hlx1 (75bp) or tandem-Hlx1 (114bp) DNA in 1x binding buffer (10mM Tris, 50mM KCl, 1mM DTT, pH7.5), supplemented with 0.05% NP-40 and 50µM ZnCl_2_. The protein was added in concentrations of 1.5µM – 2.3nM for single-Hlx1; 2.3µM – 1.5nM for tandem-Hlx1, which was prepared in a dilution series of 1:1.5. As non-specific competitor, polydIdC (Sigma-Aldrich) was added to the reactions either to a final concentration of 50ng/µl or to the highest protein concentration and was then diluted with the protein to keep the protein:polydIdC ratio to a constant level. The binding reactions were incubated for 1hr at room temperature, completed by 1x EMSA loading dye (0.5x TBE, 2.5% glycerol, 0.04% bromophenol blue, 0.04% xylene cuanol FF) and separated along a 5% polyacrylamide gel for 45 minutes at 100V. The rest of the protocol was performed according to the general EMSA protocol described in (Striedner et al., 2017).([Striedner et al., 2017](#_ENREF_63))

### Multimer Assays

In this study, different experiments were performed in order to investigate the protein stoichiometry of PRDM9.

**Extended multimer assay I.** For the multimer *assay I* (Figure 2B and Figure S3A), optimal protein and biotinylated DNA concentrations, listed in Table S10, were mixed in binding reactions supplemented by 1x binding buffer (10mM Tris, 50mM KCl, 1mM DTT, pH7.5), 50ng/µl polydIdC, 0.05% NP-40 and 50µM ZnCl_2_. In each reaction, one type of PRDM9 (eYFP-ZnF^Cst^, ZnF^Cst^, eYFP-ZnF^Cst^ 1-11, ZnF^Cst^ 1-11 or eYFP-ZnF^Cst^ 2-11) was added to a series of biotinylated single- and tandem-Hlx1 DNA fragments (single-Hlx1-75bp, tandem-Hlx1-114bp, tandem-Hlx1-232bp, tandem-Hlx1-352bp, tandem-Hlx1-468bp, single-Hlx1-740bp, single-Hlx1-856bp, single-Hlx1-1053bp, single-Hlx1-1147bp and single-Hlx1-1460bp), containing the 34bp target sequence from the murine *Hlx1* hotspot. Reactions were incubated either at 4°C overnight or for 20min at room temperature (for details see Table S10). Before loading the gel, the usDNA fragments for upper and lower reference DNA (4368bp and 220bp, respectively) were added, the reactions were then completed by 1x EMSA loading dye and separated along a 5% polyacrylamide gel for 120 minutes at 100V in a cooling box (for details see Table S10). The rest of the protocol was performed according to the general EMSA protocol described.

**DNA only.** As a control experiment, the *extended multimer* *assay I* was repeated in the exact same way except of adding a protein sample. Only the unbound DNA fragments were detected to also test for any signals coming from DNA impurities (Figure S3C).

**Small multimer assay I.** In order to perform multimer *assay I* experiments with the two shortest PRDM9 constructs, ZnF^Cst^ 2-8 and ZnF^Cst^ 2-6 (Figure S3B), optimized protein and biotinylated DNA concentrations, listed in Table S10, were mixed in binding reactions supplemented by 1x binding buffer (10mM Tris, 50mM KCl, 1mM DTT, pH7.5), 50ng/µl polydIdC, 0.05% NP-40 and 50µM ZnCl_2_. In each reaction, one type of PRDM9 was added to a series of biotinylated single- and tandem-Hlx1 DNA fragments (single-Hlx1-75bp, tandem-Hlx1-114bp, tandem-Hlx1-232bp, single-Hlx1-543bp and single-Hlx1-740bp) containing the target sequence from the murine *Hlx1* hotspot. Since those PRDM9 types have a very low molecular weight, the DNA-bound signal could not be differentiated from DNA-unbound signal on the EMSA membrane when using DNA target sequences of very high molecular weight, therefore, only five instead of ten differentially sized target molecules have been used. Reactions were incubated at 4°C overnight. Before loading the gel, the usDNA fragments for upper and lower reference DNA (2585bp and 75bp, respectively) were added, the reactions were then completed by 1x EMSA loading dye and separated along a 5% polyacrylamide gel for 60 minutes at 100V (for details see Table S10). The rest of the protocol was performed according to the general EMSA protocol described.

**Multimer assay II.** To perform multimer *assay II* experiments (Figure 2D), optimized amounts of protein and biotinylated DNA concentrations, listed in Table S10 were used in 1x binding buffer (10mM Tris, 50mM KCl, 0.05% NP-40, 50µM ZnCl_2_, pH7.5) supplemented by 50ng/µl polydIdC. As proteins eYFP-PRDM9^Cst^, MBP-eYFP-ZnF^Cst^, MBP-eYFP-ZnF^Dom2^, Halo-ZnF^Cst^ 1-11, eYFP-ZnF^Cst^ 1-11, eYFP-ZnF^Cst^ 2-11, ZnF^Cst^ 2-8 and ZnF^Cst^ 2-6 have been used for those experiments. For the PRDM9^Cst^ constructs the single-Hlx1-75bp target DNA was used, whereas for the PRDM9^Dom2^ construct a different 75bp DNA was used coming from the Pbx1 hotspot. A DNA ladder was used as a standard, by mixing 5nM single-Hlx1-75bp, 1.85nM tandem-Hlx1-114bp, 2.5nM single-Hlx1-273bp, 0.3nM single-Hlx1-543bp and 0.5nM single-Hlx1-740bp. The reactions were incubated at room temperature for 60min, completed by 3nM biotinylated usDNA-75bp for lower reference band and 1x EMSA loading dye and separated along a 5% polyacrylamide gel for 75 minutes at 100V. After 65min of electrophoresis, again 3nM biotinylated usDNA-75bp was loaded to the gel for the upper reference DNA. The rest of the protocol was performed according to the general EMSA protocol described.

**Data analysis.** Relative migration distances of the lower- and super-shift complexes, as well as of the upper and lower reference DNA molecules were measured using the Image Lab 5.1.1 software from Bio-Rad. Data evaluation was performed as described in Figure 2, Materials and Methods, Table S2 and Table S3.

### Sandwich Assay

In order to test how many DNA molecules are bound to the PRDM9 complex at the same time (Figure 4), 5nM of each DNA fragment, single-Hlx1-75bp and single-Hlx1-273bp, have been used in combination with 0.25µl SN lysate of eYFP-ZNF^Cst^ 1-11 in a 20µl binding reaction supplemented with 1x binding buffer (10mM Tris, 50mM KCl, 1mM DTT, pH7.5), 50ng/µl polydIdC, 0.05% NP-40 and 50µM ZnCl_2_. The reactions were incubated at 4°C overnight, completed by 1x EMSA loading dye and separated along a 5% polyacrylamide gel for 70 minutes at 100V. The rest of the protocol was performed according to the general EMSA protocol described.

# Supplementary_Statistical_Analysis

## Multimer Assay I

In Figure A1 we plot the value of the multimer analysis via dot-plots, stratified per PRDM9 construct. We can see differences between the groups and therefore, proceed to conduct an ANOVA to test for significant differences in the value of the multimer analysis between PRDM9 constructs.


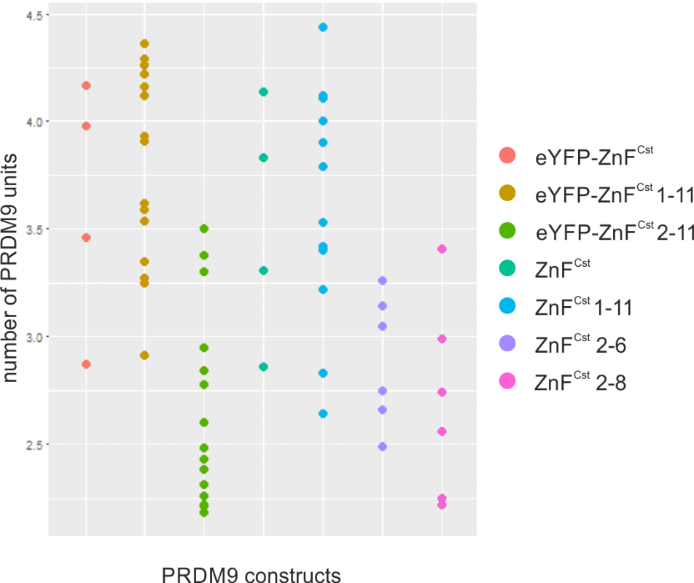


**Figure A1.** Dot-plot of multimer *assay I* for statistical analysis.

This analysis reveals significant differences (p-value < 0.00001) between the different PRDM9 constructs. After checking for normality as well as homoscedastic errors we extract all pairwise significant differences, tested via TukeyHSD-test. We list these selected significant pairwise differences in Table A1 with the computed differences, the lower and upper bounds of the confidence interval and the adjusted p-values per combination.

|  |  | diff | lwr | upr | p adj |
| --- | --- | --- | --- | --- | --- |
| **YFP-ZnF^Cst^ 2-11** | YFP-ZnF^Cst^ | -0.95 | -1.76 | -0.15 | 0.01 |
| **YFP-ZnF^Cst^ 2-11** | YFP-ZnF^Cst^ 1-11 | -1.12 | -1.64 | -0.60 | 0.00 |
| **ZnF^Cst^ 2-6** | YFP-ZnF^Cst^ 1-11 | -0.89 | -1.59 | -0.19 | 0.00 |
| **ZnF^Cst^ 2-8** | YFP-ZnF^Cst^ 1-11 | -1.09 | -1.79 | -0.39 | 0.00 |
| **ZnF^Cst^** | YFP-ZnF^Cst^ 2-11 | 0.87 | 0.06 | 1.68 | 0.03 |
| **ZnF^Cst^ 1-11** | YFP-ZnF^Cst^ 2-11 | 0.95 | 0.40 | 1.50 | 0.00 |
| **ZnF^Cst^ 2-6** | ZnF^Cst^ 1-11 | -0.73 | -1.45 | 0.00 | 0.05 |
| **ZnF^Cst^ 2-8** | ZnF^Cst^ 1-11 | -0.92 | -1.64 | -0.20 | 0.00 |

**Table A1.** Statistical analysis of multimer *assay I*.

## Multimer Assay II

We plot the values of the multimer analysis per PRDM9 constructs in Figure A2. The next step is to test the visible differences between the PRDM9-types with an ANOVA.


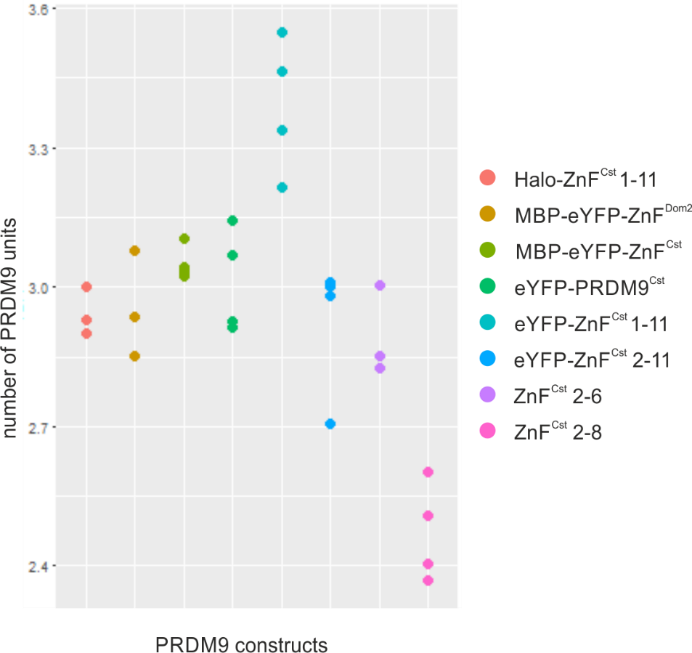


**Figure A2.** Dot-plot of multimer *assay II* for statistical analysis.

The resulting p-value is smaller than 0.0001 and indicates significant differences between the groups and we do not find deviations from normality or from homoscedasticity in the residuals. Hence, we list all pairwise differences along with the calculated differences as well as the upper and lower confidence interval thresholds in Table A2. The shown adjusted p-values are computed with the TukeyHSD-test as before.

|  |  | diff | lwr | upr | p adj |
| --- | --- | --- | --- | --- | --- |
| **YFP-ZnF^Cst^ 1-11** | Halo-ZnF^Cst^ 1-11 | 0.43 | 0.19 | 0.68 | 0 |
| **ZnF^Cst^ 2-8** | Halo-ZnF^Cst^ 1-11 | -0.49 | -0.74 | -0.24 | 0 |
| **YFP-ZnF^Cst^ 1-11** | MBP-YFP-ZnF^Dom2^ | 0.41 | 0.16 | 0.65 | 0 |
| **ZnF^Cst^ 2-8** | MBP-YFP-ZnF^Dom2^ | -0.52 | -0.76 | -0.27 | 0 |
| **YFP-ZnF^Cst^ 1-11** | MBP-YFP-ZnF^Cst^ | 0.34 | 0.09 | 0.59 | 0 |
| **ZnF^Cst^ 2-8** | MBP-YFP-ZnF^Cst^ | -0.58 | -0.83 | -0.33 | 0 |
| **YFP-ZnF^Cst^ 1-11** | YFP-PRDM9^Cst^ | 0.38 | 0.13 | 0.63 | 0 |
| **ZnF^Cst^ 2-8** | YFP-PRDM9^Cst^ | -0.54 | -0.79 | -0.29 | 0 |
| **YFP-ZnF^Cst^ 2-11** | YFP-ZnF^Cst^ 1-11 | -0.47 | -0.72 | -0.22 | 0 |
| **ZnF^Cst^ 2-6** | YFP-ZnF^Cst^ 1-11 | -0.51 | -0.76 | -0.26 | 0 |
| **ZnF^Cst^ 2-8** | YFP-ZnF^Cst^ 1-11 | -0.92 | -1.17 | -0.67 | 0 |
| **ZnF^Cst^ 2-8** | YFP-ZnF^Cst^ 2-11 | -0.45 | -0.70 | -0.21 | 0 |
| **ZnF^Cst^ 2-8** | ZnF^Cst^ 2-6 | -0.41 | -0.66 | -0.16 | 0 |

**Table A2.** Statistical analysis of multimer *assay II*.

References can be found in the main manuscript.
